# Effect of Propofol on breast Cancer cell, the immune system, and patient outcome

**DOI:** 10.1186/s12871-018-0543-3

**Published:** 2018-06-26

**Authors:** Ru Li, Hengrui Liu, James P. Dilger, Jun Lin

**Affiliations:** Department of Anesthesiology, Stony Brook University Health Sciences Center, Stony Brook, NY 11794-8480 USA

**Keywords:** Breast Cancer, Propofol, Proliferation, Migration, Natural killer cell

## Abstract

Breast cancer is the second leading cause of cancer death in women. Surgery is the first line of treatment for breast cancer. Retrospective clinical studies suggest that the type of anesthesia administered during oncological surgery may influence patient outcome. Propofol, the widely used intravenous anesthetic agent, may lead to better outcomes compared to volatile anesthetics. Here we review the literature on the effect of propofol in breast cancer cells, the immune system, pain management, and patient outcomes. Evidence from the study of breast cancer cell lines suggests that high concentrations of propofol have both anti-tumor and pro-tumor effects. Propofol and volatile anesthetics have different effects on the immune system. Propofol has also been shown to reduce the development and severity of acute and chronic pain following surgery. Although a retrospective study that included many types of cancer indicated that propofol increases the long-term survival of patients following surgery, the evidence for this in breast cancer is weak. It has been shown that Propofol combined with paravertebral block led to change of serum composition that affects the breast cancer cell behaviors and natural killer cell activity. Prospective studies are in progress and will be finished within 5 years. The existing evidence is not sufficient to warrant changes to current anesthetic management. Further research is needed to clarify the mechanisms by which propofol affects cancer cells and the immune system.

## Background

Breast cancer is the most common malignancy in women, and the second leading cause of cancer mortality worldwide after lung cancer. In 2017, it is estimated that 252,710 women will be diagnosed with invasive breast cancer, and 40,610 women will die of breast cancer [1]. More than 90% of women diagnosed with early stage (I or II) breast cancer have mastectomy or breast-conserving surgery, and over 70% of women with advanced breast cancer (stage III or IV) have these surgeries [2]. Surgical resection of solid breast tumors is the primary and most effective treatment. However, local or metastatic recurrence after surgery does occur and is a major cause of cancer death. Localized and regionalized breast cancer patients have more than 80% 5-year survival rate, but breast cancer patients with distant organs only have 27% 5-year survival rate [3]. In fact, the perioperative period presents many risks to patients. The process of surgery inevitably induces a profound stress response, which suppresses cell-mediated immunity, and enhances tumor growth and spread [4, 5]. This is also a growing recognition that the anesthetics used during mastectomy may affect the long-term outcome.

Propofol is the most extensively used intravenous anesthetic for induction and maintenance of anesthesia in the United States. Volatile anesthetics such as isoflurane, sevoflurane and desflurane are also in common use. The two classes of anesthetics have different effects on immune function [6]. The choice of anesthetic used during cancer surgery may affect patient outcome. A recent retrospective study found significantly better long-term survival rates for patients receiving propofol (3714 patients, 504 deaths, 13.5%), compared to patients receiving volatile anesthetics (3316 patients, 796 deaths, 24%), following cancer surgery between 2010 and 2013 at a teaching hospital in the United Kingdom [7].

In this article, we review the effects of propofol on breast cancer cell biology, the immune system, and postoperative pain. We discuss recent retrospective studies and upcoming prospective studies on the influence of propofol on long-term outcomes of breast cancer patients. The literature in this review was obtained by searching PubMed© with search terms “propofol” and “breast cancer” on December 3, 2017, and limited to articles written in English.

## Effects of propofol on breast cancer cells

Although we usually refer to breast cancer as a single disease, it includes up to 21 diverse histological subtypes, which respond distinctly to treatments and lead to various outcomes [8, 9]. Based on the presence or absence of hormone receptors (estrogen receptor/ER, or progesterone receptor/PR), and excess levels of human epidermal growth factor receptor 2 (HER2+/HER2-), breast cancer is stratified into four major molecular subtypes, including luminal A (ER+ and/or PR+/HER2-), luminal B (ER+ and/or PR+/HER2+), HER2 overexpressing (ER-/PR-/HER2+), and basal-like or triple negative (ER-/PR-/HER2-) [10, 11]. To understand the biology of breast cancer, breast cancer cell lines have been established and widely used to investigate the effect of drugs on cancer cell proliferation, apoptosis, migration and invasion. To date, more than 51 breast cancer cell lines have been separated from primary tumor, and successfully cultured in vitro [12]. To mimic the heterogeneity of breast cancer, each molecular subtype mentioned above has several representatives. For instance, MCF7 is one of the most intensively studied cell lines from luminal A subtype. MDA-MB-468 and MDA-MB-231 both belong to triple negative breast cancer cell lines. MDA-MB-231 is further classified into a subgroup named “claudin-low” which signifies low gene expression of tight junction proteins claudin3, 4, 7 and E-cadherin.

As the number of tumor surgeries performed under general anesthesia increases, the hypothesis that propofol has direct effects on cancer cells has become attractive. Indeed, a number of studies have shown that propofol influences the function of breast cancer cells and may do so via various cellular pathways.

### Anti-tumor effects

Li et al. found that propofol (2–10 μg/mL) significantly suppressed migration and invasion of MDA-MB-231 cells [13]. They demonstrated that propofol reduced the expression and secretion of matrix metalloproteinase (MMP) -2 and − 9. MMPs are thought to promote cancer cell invasion and metastasis by degrading extracellular matrix proteins. The effect of propofol may be due to attenuation of the phosphorylation of NF-κB and IKK. IKK is a kinase degrades IkB, which normally leads to NF-κB activation. Thus, propofol interferes with NF-κB pathways in at least two ways.

Ecimovic et al. examined the effects of propofol (1–10 μg/ml) on two breast cancer cell lines: MDA-MB-231 and MCF7, which are ER- and ER+ respectively. They found that propofol impaired migration but not proliferation of both cell lines. This was mediated by decreased expression of neuroepithelial cell transforming gene 1 (NET1) [14]. NET1 has also been associated with promotion of migration in adenocarcinoma in vitro [15].

A recent study showed that propofol downregulated miR-24 and increased p27 expression and cleaved caspase-3 expression in MDA-MB-435 cells, all of which lead to induced cell death [16]. MDA-MB-435 is a cell line originated from a metastatic breast cancer but its origin could be melanoma instead [17].

Conjugation of fatty acids docosahexaenoic and eicosapentaenoic acid to propofol result in compounds that suppress cell adhesion, migration, and induce apoptosis more effectively than propofol itself. This was observed in several breast cancer cell lines MDA-MB-231, MCF-7 (ER^+^) AU565 (Her-2^+^) and MDA-MB-361 (ER^+^, Her-2^+^) [18, 19]. The fatty acids alone were not effective. The mechanism for this is not clear but may involve inhibition of histone deacetylase activity. It is also not known whether these propofol conjugates are anesthetics.

**Fig. 1:**
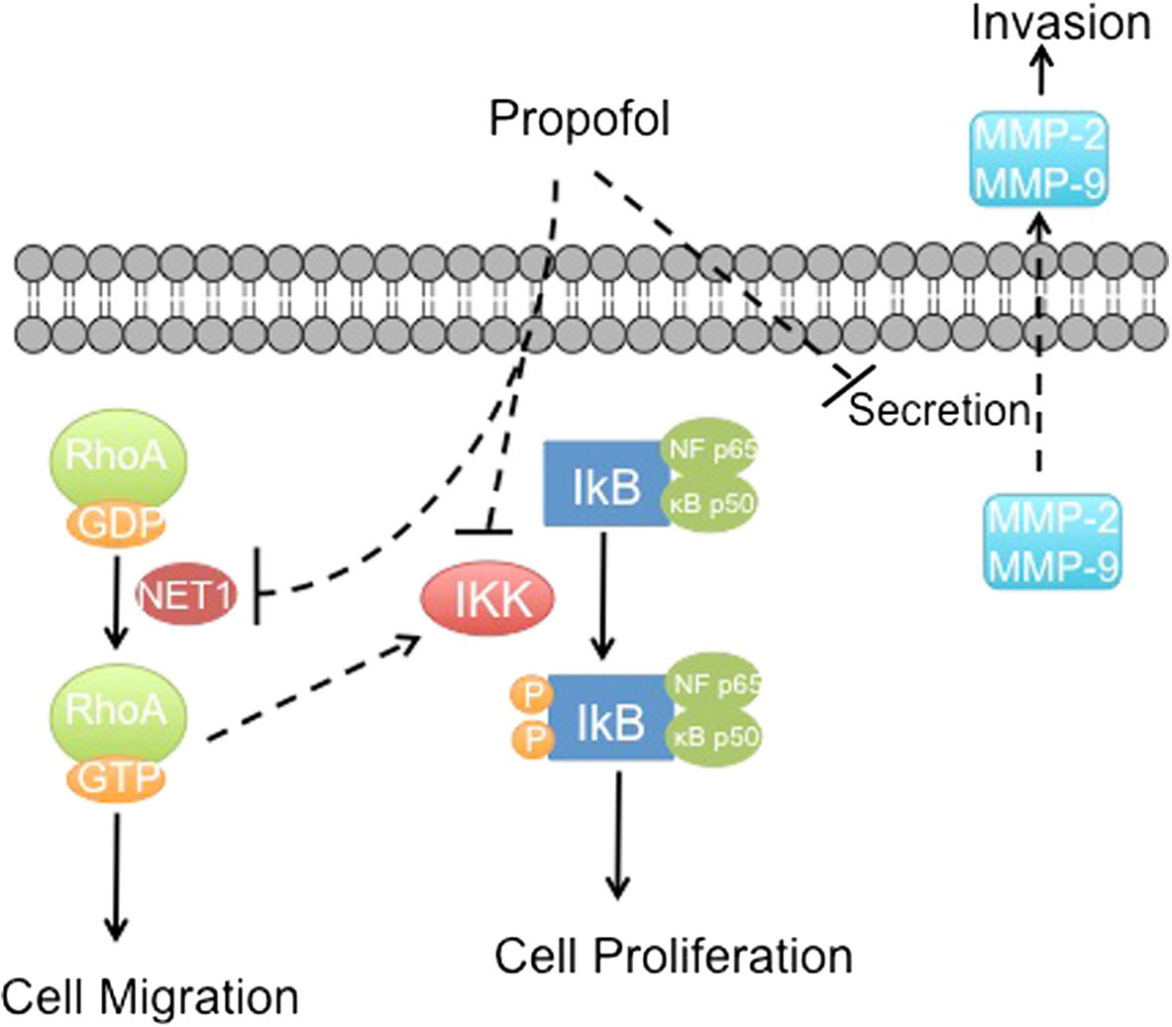
Proposed mechanisms for the anti-tumor effects of propofol. Propofol impairs cell proliferation through inhibition of NF-kB pathway. It also reduces cell migration and invasion by inhibiting NET1, and decreasing the secretion of MMP2/MMP9

The in vitro studies described above presume that there is a direct effect of propofol on breast cancer cells. We summarized the potential mechanism of the direct anti-tumor effects of propofol in Fig. 1. Moreover, it is also possible that the effect of propofol is indirect. Several studies have used the serum from breast cancer patients who received one or another type of anesthesia. A group of patients was randomized to receive either propofol/paravertebral anesthesia-analgesia or sevoflurane general anesthesia with opioid analgesia. It should be noted that the addition of a paravertebral block with local anesthetics reduces the requirement of general anesthetics and opiates by blocking the afferent pain and sensory transmission, and suppressing the efferent sympathetic discharge that affect the immune function. Thus, it may be difficult to isolate the effects of propofol from those of the paravertebral block.

The first study examined proliferation and migration of MDA-MB-231 cells in the presence of 2–10% of serum obtained from patients 24-h post-op. Cancer cell migration was not significantly affected by serum from patients receiving either propofol/paravertebral block or sevoflurane/opioid anesthesia. Incubation of cells with 10% serum from patients in the propofol/paravertebral group resulted in decreased viability compared to that from serum from patients receiving sevoflurane/opioid anesthesia. This decrease in viability is probably due to increased apoptosis [20].

In the second study, ER+/PR+ breast cancer cells (HCC1500) were co-cultured, for 24 h, with nature killer (NK) cells obtained from healthy donors in the presence of serum from patients receiving either propofol/paravertebral or sevoflurane/opioid anesthesia. The serum from the propofol/paravertebral group significantly induced apoptosis of HCC1500 cells, but the serum from sevoflurane/opioid group did not. Concomitantly, the serum from propofol/paravertebral patients increased the expression of the surface protein CD107a on NK cells without alteration of normal NK marker expression or secretion of cytokines [21]. CD107a is upregulated in stimulated NK cells and this correlates with NK cell-mediated lysis of target cells [22]. Serum from the sevoflurane patient reduced NK cell activating receptor CD16, IL-10, IL-1β, all thus lowering the activity level of NK cells [22, 23].

### Pro-tumor effects

In 2002, Garib et al. observed that propofol (3–9 μg/mL) increased migration of MDA-MB-468 cells [24]. They also found that propofol increased intracellular calcium and altered the organization of the actin cytoskeleton of these cells [25]. These effects of propofol were inhibited by the L-type calcium channel blocker verapamil. The group tested the hypothesis that propofol was acting via GABA-A receptor chloride channels. Although they found evidence for the gamma subunit of the GABA-A receptor and the effect of propofol was inhibited by the GABA-A antagonist bicuculline, they did not provide evidence for functional channels in the cells. It should be noted that bicuculline is not specific for GABA-A receptor channels; it is also known to block small conductance Ca^++^-activated K^+^ channels at similar concentrations [26].

In a recent study, treatment of MDA-MB-231 cells with propofol (2–10 μg/mL) for up to 12 h was shown to increase cell proliferation and migration. The functional changes in the cells were correlated with an increase in nuclear factor E2-related factor-2 (Nrf2) expression level, and a decrease in p53 expression level, caspase-3 activity and percentage of apoptotic cells [27].

**Fig. 2:**
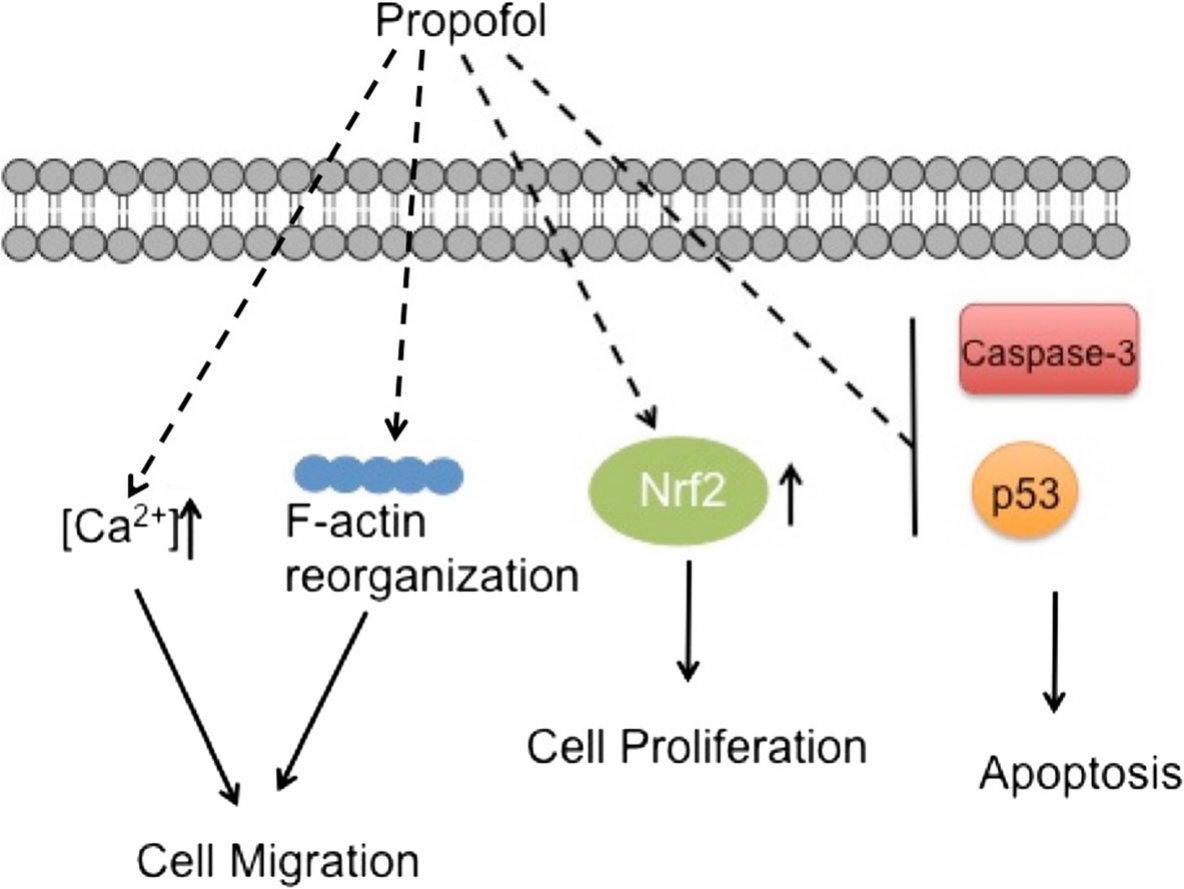
Proposed mechanisms for the pro-tumor effects of propofol. Propofol enhances migration of breast cancer cells by increasing intracellular Ca^2+^ level and rearrangement of F-action. Propofol also promotes cell proliferation through Nrf2 pathway, and inhibits apoptosis by decreasing the expression of p53 and caspase-3

In summary, most studies conclude that propofol suppresses cancer cell viability and migration (Table 1). Some results are inconsistent with this though. The potential mechanism of pro-tumor effects is summarized in Fig. 2. One possible explanation for this discrepancy is the heterogeneity of breast cancer: propofol may have different actions on different cancer cell types. Another factor is non-standardization of experimental parameters such as the concentration and time of exposure to anesthetic. So far, there are few insights into the mechanism of action of propofol. Propofol has been identified to modulate HIF-1α pathway in prostate cancer cells, which may shed light to the analysis of molecular mechanism of propofol in breast cancer [28]. Again, the heterogeneity of breast cancer may be obscuring a consistent mechanism for propofol. With the rapid advances in system biology, the mechanism of anesthetic-induced effects on cancer cells could be explored by using various “omics” technology. On the other hand, cancer cell lines could only reflect part of the overall impact of propofol on cancer, as propofol may affect the progression of cancer in many other aspects.

**Table 1:** Summary of the studies that examined direct effects of propofol on breast cancer cells

Study	Cell line	[Propofol] (μg/ml)	Incubation time (hr)	Proliferation (method)	Migration (method)	Invasion (method)
Li et al	MDA-MB-231	2–10	24		↓ (wound healing)	↓ (Matrigel membrane)
Ecimovic et al	MDA-MB-231	6–10	24	No change (MTT)	↓ (8 μm pores)	No change (Matrigel membrane, 6 h)
Ecimovic et al	MCF7	4–10	24	No change (MTT)	↓ (8 μm pores)	↓ (Matrigel membrane, 6 h)
Yu et al	^a^ MDA-MB-435	2 (10 μM)	6	↓ (TUNEL stain)		
Siddiqui et al	MDA-MB-231	20 (100 μM)	24	↓ (WST-1)	No change (8 μm pores, 4 h, 5 μg/ml)	
Garib et al	MDA-MB-468	3–9	10		↑ (collagen matrix, video tracking)	
Meng et al	MDA-MB-231	2–10	12–24	↑ (MTT, 10 h)	↑ (wound healing)	

Note that propofol concentration and/or incubation time in a study may be different for different assays. This is noted in the Migration and Invasion columns

^a^MDA-MB-435 is a cell line originated from a metastatic breast cancer but its identity could be melanoma instead

One must use caution in interpreting the results of in vitro cell culture experiments. Most of the studies cited in this review used concentrations of propofol in the range of clinical doses (2 to 4 μg/mL [29–31]). However, propofol binds extensively to erythrocytes (50%) and serum proteins almost exclusively albumin (48%) [32]. As a result, cancer cells (and all other types of cells) in vivo are exposed to < 2% of the total propofol concentration. In contrast, the standard cell culture medium typically containing 10% fetal bovine serum used in in vitro experiments contains a much lower level of serum albumin (about 0.17 ~ 0.34 g / dL) and negligible erythrocyte than that found in human plasma (3.5 ~ 5.0 g / dL albumin and abundant erythrocytes). Because of this, about 95% of the total propofol in a cell culture dish is free to bind to the cancer cells. Therefore, the free propofol concentration employed in the in vitro cell culture experiments may be 50–500 times that of the clinical concentrations. This would be a lethal dose for humans. Future in vitro experiments should be designed to take protein binding into account.

## Propofol and the immune system

Surgical trauma is associated with an increase of cytokines and stress hormones in the plasma during the vulnerable peri-operative period [17]. This stress response induces transient suppression of cell-mediated immunity, the primary immune-defense against the invasion of tumor cells and micro-metastasis [33]. Surgery and anesthesia could suppress the immune response by activating the hypothalamic-pituitary-adrenal axis and the sympathetic nervous system [34]. Anesthetics may also directly affect the functions of immune cells, including natural killer (NK) cells, cytotoxic T cells, mononuclear cells and dendritic cells [35, 36]. NK cells are large, granular cytotoxic lymphocytes that naturally recognize malignant cells, and induce lysis without prior sensitization. Low perioperative levels of NK activity are associated with an increased cancer related morbidity and mortality in humans [37]. Melamed and colleagues demonstrated in rats that ketamine, thiopental, and halothane but not propofol reduced the number of circulating NK cells and depressed NK cell cytotoxicity [38]. As discussed in the previous section, serum from patients who received sevoflurane/opioid anesthetics impaired NK cell induced lysis of breast cancer cells, while propofol-paravertebral anesthesia did not [21]. The same group also demonstrated that propofol-paravertebral anesthesia increased the infiltration of NK and T helper cells into breast cancer tissue [39]. A recent prospective randomized study measured the NK cell cytotoxicity in fifty patients 24 h after breast cancer surgery. Patients were randomly assigned to receive either propofol-remifentanil anesthesia with postoperative ketorolac analgesia or sevoflurane-remifentanil anesthesia with postoperative fentanyl analgesia. Propofol-ketorolac treatment increased NK cell cytotoxicity by 30% (*p* = 0.048) compared to baseline whereas there was a 16% decrease in the sevoflurane-fentanyl group (*p* = 0.032) [40]. During stress conditions, the sympathetic nervous system releases catecholamines which suppress NK activity through β-adrenergic stimulation and therefore promote metastasis [41]. Propofol is known to interfere with β-adrenergic signal transduction in adipocytes [42]. Thus, the favorable impact of propofol on NK cells may be partially explained through the mechanism of β-adrenergic stimulation.

## Propofol and postoperative pain management

Another risk factor associated with cancer surgery is postoperative acute and chronic pain. There is evidence that effective perioperative analgesia attenuates surgery-induced metastasis; this is likely due to a reduction in the stress response [43]. However, opioid-based analgesia has been implicated in potentiating tumor cell survival and angiogenesis [44], thus alternative strategies have been explored to limit the use of opioid. Two clinical studies have suggested that propofol may reduce the development and severity of both acute and chronic postoperative pain. The first retrospective study was conducted with 175 women 3 to 4 years after breast cancer surgery. Patients in the sevoflurane group (*n* = 89) were more likely to have chronic pain 2 ~ 24 months after surgery in comparison with propofol group (*n* = 86) (*p* = 0.007). Among patients with chronic pain from both groups, the choice of anesthetics made no difference in the severity or duration of the pain [45]. The second study prospectively randomized 66 patients scheduled for mastectomy to receive thoracic paravertebral blocks with either propofol-based total intravenous anesthesia or sham subcutaneous local anesthetic injections with sevoflurane-based general anesthesia. The paravertebral blocks with propofol significantly reduced the 6-mouth chronic neuropathic pain (CNP) risk according to the DN4 questionnaire, a screening tool, and the CNP grading system defined by International Association for the Study of Pain. The effect could be attributable to either the vertebral block, propofol or both [46].

## Propofol and clinical trials in breast cancer

Three retrospective studies have compared propofol with volatile anesthetic agents on clinical outcomes of patients undergoing breast cancer surgery (Table 2). The first study, published in 2014 by Enlund and colleagues, reviewed surgical data from 1837 breast cancer patients with 620 in the propofol group and 1217 in the sevoflurane group. Differences in overall survival rates (propofol vs sevoflurane) for breast cancer were 3% (95% confidence interval, 1 to 4%, *p* < 0.001) at 1 year, and 2% (− 2 to 6%, non-significant) at 5 years, in favor of propofol. However, the observed differences were eliminated after adjustment for confounders (history of cardiac ischemia was 1.8 times more likely in the sevoflurane group) [47]. The second study analyzed 325 patients with 173 in the propofol group and 152 in the sevoflurane group. No difference was observed in the overall 5-year survival rate between two groups, although the propofol group did have a lower rate of cancer recurrence (*p* = 0.037) [48]. The third study collected 2645 cases, which included 2589 in the volatile anesthesia group and 56 in the propofol group. There was no significant difference between the groups in terms of postoperative recurrence or survival probability [49]. Taken together, these studies suggest that propofol may either improve overall survival rates or lower the rate of cancer recurrence, but the evidence is not overwhelming. It is good to consider the limitations of these studies. First, there is the inherent limitation of retrospective studies in that they do not standardize clinical care and do not randomize patient groups. Therefore, the effects of confounding factors and selection bias cannot be easily eliminated. Second, one of the studies (Lee et al. [49]) had a relative small patient population and another (Kim et al. [50]) had an extremely uneven distribution between the two anesthetic groups.

**Table 2:** Clinical trials comparing volatile anesthetics with propofol in breast cancer surgery

Type of Study	Reference	Cancer	Surgery Type	Anesthetic Technique	Number of Patients	Outcome (95% CI)	Estimated Completion Date
Retrospective	Enlund et al.	Breast Cancer	Breast cancer surgery	GA with propofol or sevoflurane	1873 (620 in propofol and 1217 in sevoflurane)	3% higher for overall survival at one year in propofol group (p < 0.001)	Finished
Retrospective	Lee et al	Breast Cancer	Modified radical mastectomy	Propofol-based TIVA or sevoflurane-based anesthesia	325 (173 in TIVA and 152 in sevoflurane)	Propofol group showed a lower rate of cancer recurrence (*p* = 0.037)	Finished
Retrospective	Kim et al	Breast Cancer	Breast Cancer surgery	Propofol-based TIVA or sevoflurane-based anesthesia	2645 (56 in TIVA and 2589 in volatile--base anesthesia)	No difference	Finished
Prospective	University of Zurich	Breast Cancer	Breast Cancer surgery	GA with propofol TCI or sevoflurane	231	Circulating tumor cells at 3 days after surgery	August, 2017
Prospective	Konkuk University Medical Center	Breast Cancer	Breast Cancer surgery	GA with propofol or sevoflurane	300	NK cell activity at 1 and 24 h after surgery	July, 2020
Prospective	Uppsala University	Breast, colonic and rectal cancer	Breast Cancer surgery	GA with propofol or sevoflurane	2000	Overall 5-year survival	December, 2022

*GA* general anesthesia, *TIVA* total intravenous anesthesia, *TCI* target-controlled infusion, *NK* cell natural killer cell

To obtain hard evidence of a causal relationship between anesthetics and cancer outcomes, prospective randomized controlled trials are needed. There are six current prospective randomized clinical trials registered on databases worldwide comparing volatile anesthetics to propofol anesthesia [50]. Three of them specifically focus on or include breast cancers (Table 2). The first study is analyzing the number of circulating tumors cells at 3 days after surgery [51]. The primary outcome of the second study is NK cell activity at 1 and 24 h after surgery [52]. Those two studies may further provide translational evidence on mechanism of propofol on cancer outcome. In the third study, the primary endpoint is the 5-year survival rate [53]. All three studies compare the volatile anesthetic sevoflurane with propofol.

## Conclusion

For many years, the relationship between anesthetic techniques and cancer outcomes has captured interest in oncological surgery. The current literature suggests that the choice of anesthetic is correlated with cancer patient survival or recurrence after surgery with propofol considered beneficial compared to volatile anesthetics. The potential benefits of propofol might include impairment of cancer cell functions, preservation of immune function and reduction in surgical stress. However, the existing laboratory studies and clinical trials including propofol are inconsistent and inconclusive. Most in vitro studies focus on the analysis of changes in cancer cell behavior in response to propofol but do not delve deeply into potential mechanism. The beneficial effects of propofol on immune system are largely restricted to NK cells. Moreover, most clinical studies are retrospective, which are not sufficient to recommend changes in anesthesia practice. Hopefully the ongoing prospective clinical trials may offer a more definite answer. In the meantime, additional studies are required to help unravel the role anesthetics in the immune function using animal models, and explore potential therapy to counteract the dentrimental effect of certain anesthetics. In terms of direct effect of propofol on cancer cell, protein/gene targets are necessary to decipher the molecular mechanism, particularly with respect to the heterogeneity of breast cancer. Additional in vivo and in vitro studies are urgently needed to improve our current clinical guidance.

## Abbreviations

CNP: Chronic neuropathic pain
ER: Estrogen receptor
GA: General anesthesia
GABA-A: Type A gamma-aminobutyric acid receptor
HER2: Human epidermal growth factor receptor 2
HIF-1α: hypoxia-inducible factor-1 alpha
IKK: I kappa B kinase
MMP: Matrix metalloproteinase
NET1: Neuroepithelial cell transforming gene 1
NF- κB: Nuclear factor kappa-light –chain-enhancer of activated B cells
NK cell: Nature killer cell
Nrf2: E2-related factor-2
PR: Progesterone receptor
TCI: Target-controlled infusion
TIVA: Total intravenous anesthesia
